# *Dillenia* (Dilleniaceae) pollen heteromorphy and presentation, and implications for pollination by bats

**DOI:** 10.1002/ece3.10997

**Published:** 2024-02-09

**Authors:** Sophie Petit, Annette T. Scanlon, Alivereti Naikatini, Tara Pukala

**Affiliations:** ^1^ UniSA STEM University of South Australia Mawson Lakes South Australia Australia; ^2^ NatureFiji‐MareqetiViti Suva Fiji; ^3^ Kangaroo Island Research Station Penneshaw South Australia Australia; ^4^ Department of Primary Industries and Regions Invasive Species Unit, Biosecurity Adelaide South Australia Australia; ^5^ Research and Development Division Ministry of Forestry and Fisheries, Fiji Government Suva Fiji; ^6^ School of Physics, Chemistry and Earth Sciences University of Adelaide Adelaide South Australia Australia

**Keywords:** anther opening, heteranthery, Melastomataceae, pollen morphology, Pteropodidae, staminodes

## Abstract

Bat pollination of *Dillenia* in Fiji, a genus that was presumed to be pollinated by bees, posits that other *Dillenia* species may be bat‐pollinated, with implications for conservation and the understanding of angiosperm evolution. Botanical descriptions of some corolla behaviours (‘falling as a whole’) suggest bat removal of permanently closed corollas, as in *D. biflora*. Considering the remoteness of species of interest, we reviewed some *Dillenia* floral traits to hypothesise what they may mean for bat pollination of the genus. We investigated *D. biflora* pollen grains apertures and reviewed *Dillenia* literature concerning corolla behaviour and colour, and pollen apertures and presentation, including pores and staminodes. Our samples had dramatically different ratios of tricolpate to tetracolpate pollen grains, a trait that does not exclude pollination by bees. Petal colour polymorphism occurs, with mixed colours proportionately less common in flowers with corollas that open. The proportion of species with staminodes did not differ between those presumed to be pollinated by bats and others, but anthers of the former were significantly more likely to have apical pores, and stamens all had similar length or were slightly longer in the middle, whereas stamens in two distinct groups occurred in 55% of bee‐pollinated species. Pollen heteromorphy may facilitate pollination by different taxa in tropical environments. However, anther apical pores and stamen uniformity are more likely to be associated with bat‐pollinated species than are other morphologies. *Dillenia* could be a useful model to examine evolutionary aspects of colour, heteranthery, staminodes and pollen heteromorphy. Only field work will verify bat pollination and the implications of bat dependence for *Dillenia* species.

## Graphical abstract

*Dillenia* species, like all members of the phylogenetically mysterious family Dilleniaceae, were believed to be nectarless and pollinated by bees until the discovery that *D. biflora* was pollinated by bats in Fiji. Uniform stamens and apical anther pores, dominant traits in species suspected to be pollinated by bats because their corollas do not open, add evidence that several *Dillenia* species, including threatened ones, may need bats for pollination.

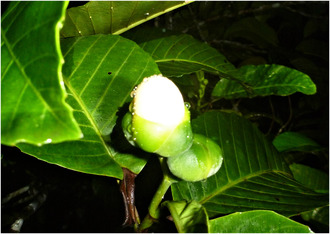

## 1 INTRODUCTION

The phylogenetic placement of Dilleniaceae ‘remains one of the last major mysteries in the angiosperms’ (Soltis et al., 2018). The recent discovery of a novel pollination system (chiropteropisteusis) involving the obligate removal of permanently closed *Dillenia biflora* (A.Gray) Guillaumin petals by bats for pollination has large implications for the understanding of flowering plant evolution and the conservation of the genus *Dillenia* (Dilleniaceae) across its range (Petit et al., 2022). The large and stout flowers of *Dillenia* spp. were all initially believed to be nectarless and buzz‐pollinated by bees (Endress, 1997; Horn, 2007; Soltis et al., 2018), but *D. biflora* contains abundant nectar and its pollination requires bats, which brings a new element to the study of the evolution of flowering plants (Petit et al., 2022). The benefits to plants of specialising on bat pollinators include abundance and diversity of pollen dispersed over long distances (Fleming et al., 2009).

The descriptions of flower corolla behaviours by Hoogland (1952, 1959, ‘corolla falling as a whole without spreading’ or ‘petals falling off without spreading’) suggest that several *Dillenia* species other than *D. biflora* are pollinated by bats; at least 10 species are concerned, including three threatened ones (Petit et al., 2022). The World Flora Online (2022) recognises 68 *Dillenia* species. They are widely distributed from the southern Himalayas, southwest China and Hainan to the northeast coast of Queensland in Australia and from eastern Madagascar to Fiji (Hoogland, 1952; Horn, 2007). Conservation of chiropteropisunous (bat‐dependent) species is concerning considering the high level of threat facing island bats (Vincenot et al., 2017), including in Fiji, where *D. biflora* occurs, and where cave‐dwelling blossom bat pollinators (*Notopteris macdonaldi*) are disturbed and hunted (Scanlon, Petit, & Bottroff, 2014). Several potential *Dillenia* bat pollinators identified by Petit et al. (2022) have declining populations. The dependence of bats on *Dillenia* spp. should also be of concern. For example, in Fiji, 70% of the Vulnerable *N. macdonaldi*'s bat diet samples contained *D. biflora* pollen (Scanlon, Petit, Tuiwawa, & Naikatini, 2014).

We aimed to identify whether some *Dillenia* floral traits may hint at pollination by bats. The three main categories of floral traits linked to pollination are fit (efficiency), signal (inflorescence size, colour, scent) and reward (Sletvold, 2019). The mechanical fit between flowers and pollinators influences pollen competition and seed set. For example, the bodies of pollinators acting as male–male competition ‘arenas’ may affect pollen transfer efficiency and floral evolution (Minaar & Anderson, 2021; Minnaar et al., 2019; Opedal et al., 2023). Bilateral symmetry, fused corollas, horizontal orientation and reduced stamen numbers improved the precision of pollen placement on honeybees and bats in Thailand (Stewart et al., 2022). Pollen presentation in different *Dillenia* species could affect bat pollination success. Signals and rewards also affect pollen transfer by influencing pollinators. *Dillenia* species offer a range of petal colours, which could affect discrimination by bats since members of the Pteropodidae have great colour perception (e.g., El‐Mansi et al., 2020) as well as scent perception (e.g., Raghuram et al., 2009). Regarding rewards, the main bat rewards are nectar and pollen (Barclay, 2002; Law, 1992). Only *D. biflora* is known to produce nectar so far among *Dillenia* species (Petit et al., 2022). A few plant species reward bats with flower petals or bracts (e.g., Cox, 1984; Nathan et al., 2009); for example, the removal of the fleshy and sweet staminodial tube is necessary for female flower pollination in the palm *Calyptrogyne ghiesbreghtiana* (Cunningham, 1995; Tschapka, 2003). By reviewing the literature, which is scant on *Dillenia* pollen and pollination, but offers information on petal colour and pollen morphology and presentation, we aimed to determine how floral traits including pollen apertures and presentation may be associated with presumed bat pollination in *Dillenia*.

Although the association of particular floral structures with bat pollination is well known (Domingos‐Melo et al., 2023; Faegri & van der Pijl, 1979; Fleming et al., 2009; Willmer, 2011), that of pollen morphology and pollen presentation is much less so. Intraspecific differences in pollen traits are associated with different pollinators (Hasegawa et al., 2023). In their review for caesalpinioid legumes, Banks and Rudall (2016) associated bat pollination with some degree of pollen supraectal ornamentation, as was also suggested by Dos Santos et al. (2012). Yang et al. (2020) observed a strong relationship between pilate, striate and fossulate ornamentation and bat pollination in lamiids, although they had few samples. Stroo (2000) found that large size of pollen was the only trait that characterised bat‐pollinated plants. They had variable numbers of pollen apertures among families, and rough exine also tended to be associated with bat‐pollinated species (Stroo, 2000). No species appeared to have heteromorphic pollen. How the number of pollen apertures may be associated with bat pollination is unclear for heteromorphic species, but this trait does not seem rare in taxa that are in part pollinated by bats (e.g., Sapotaceae, Harley, 1991; Sapindales, Gonçalves‐Esteves et al., 2022). The pollens of six of 11 *Bauhinia* species (Leguminosae, Caesalpinioideae) in Brazil's Caatinga varied in aperture number (Dos Santos et al., 2012), including *B. pentandra*, which is pollinated by bats (Domingos‐Melo et al., 2023).

Eudicots are known as mostly tricolpate in recognition of the three colpi (bearing three furrow‐like apertures; Walker & Doyle, 1975) of their pollen grains, although some magnoliids may also be tricolpate (Doyle & Hotton, 1991). A larger number of apertures, by increasing the potential number of germination sites, may be advantageous (Furness & Rudall, 2004). Species with aperture polymorphism occur (heteromorphy within individual, and rarely, polymorphism among individuals; Mignot et al., 1994). Pollen morphology in Dilleniaceae is diverse, including within a sample (presumed to be individual plant; Dickison et al., 1982). Pollen heteromorphy may facilitate pollination in variable environments and is thus an interesting trait to examine in relation to bat pollination. In *Viola diversifolia*, more apertures resulted in faster pollen germination, but a lower rate of tube growth and life expectancy (Dajoz et al., 1993). In contrast, using *Arabidopsis thaliana* polymorphic mutants with different numbers of apertures, Albert et al. (2018) showed that triaperturate pollen grains performed better than those with more apertures in most in vivo experiments, including longevity, but also germination. However, the ploidy of their pollen types differed. They attributed the prevalence of triaperturate pollen in the eudicots to the best combination of germination capacity, longevity and capacity to accommodate changes in volume. Pollen apertures are associated with several factors that may affect pollen performance, such as contact with stigma for pollen tube growth (but see Edlund et al., 2017), hydration/desiccation and accommodation of shrinkage and swelling (Edlund et al., 2004), with additional structure assisting with elasticity (Volkova et al., 2013). Shrinking and expansion during pollen–stigma interactions and during pollen dispersal were shown in *A. thaliana*, for which increased number of apertures increased osmotic stress (Prieu et al., 2016). Vulnerability to desiccation and potential fungal invasion (Edlund et al., 2004) may be affected in the rainforest by the mechanical resilience of pollen grains. In view of the high level of moisture in the environment, but potential desiccation from travel, aperture heteromorphy in the presumed tricolpate *D. biflora* could be a strategy to maximise long‐distance pollination success in a rainforest environment.

The presentation of pollen on stamens and in anthers should facilitate its access and transport by pollinators. It is assumed that poricidal anthers are adapted for buzz pollination by vibration‐producing bees (Buchmann, 1983; Vallejo‐Marín, 2019), but a vast analysis of the Neotropical tribe Merianieae (Melastomataceae) showed that the buzz pollination system shifted in some cases to a mixed‐vertebrate syndrome (hummingbirds, bats, rodents, flower piercers – i.e., nectar‐consuming passerine birds) with floral adaptations including a crumpled and soft thecal wall and mostly apical pore that resulted in a salt‐shaker pollen expulsion mechanism (Dellinger, Chartier, et al., 2019; Dellinger, Pérez‐Barrales, et al., 2021; but see also shift to generalised insect pollination via nectar production in de Brito et al., 2017). The position of anther pores in *Dillenia* could be a clue pointing at the salt‐shaker mechanism described by Dellinger, Chartier, et al. (2019), by which simple pressure applied by vertebrates releases the pollen grains. Evidence indicates that the shift from ancestral buzz pollination by bees to more efficient pollinators was driven by a mountain climate (Dellinger, Pérez‐Barrales, et al., 2021); bee pollination is associated with stronger genetic isolation than is pollination by flying vertebrates (Dellinger et al., 2022).

The presence of nectar is a defining proof of ‘mixed‐vertebrate’ pollination syndrome (sensu Dellinger, Chartier, et al., 2019). Although *Dillenia* species other than *D. biflora* are not yet known to produce nectar, we hypothesise that all species with closed corollas at maturity do, since they would require a strong animal, most probably equipped with teeth, to remove the corolla. Bats are the most likely candidates and require a nectar reward.

We aimed to determine whether heteranthery, or lack thereof, may be indicative of bat pollination. Heteranthery, which refers to the presence of different stamen morphologies and functions in the same flowers, is typically associated with bee‐pollinated plants (Vallejo‐Marín et al. (2010) and references therein), although it also occurs in some bird‐rewarding flowers in the Merianieae (Melastomataceae) (Dellinger, Artuso, et al., 2021). Heteranthery is generally explained by ‘division of labour’ where some anthers specialise in reproduction and others in feeding pollinators, but staggered pollen release for birds has also been described as a function of heteranthery (Dellinger, Artuso, et al., 2021), and stamen dimorphism affected some Melastomataceae pollen and stamen traits that may also have an impact on their functional role (Trevizan et al., 2023). Heteranthery can be used as a predictor in a passerine bird pollination syndrome (e.g., *Meriania macrophylla*, Valverde‐Espinoza et al., 2021). The relative rarity of heteranthery, except in the Leguminosae and Melastomataceae, was explained by the fact that pollen consumption may not be excessive or pollen placement in the ‘division of labour’ would require pollinators large enough for the pollen to be positioned differently (Vallejo‐Marín et al., 2010). We expected that bats, seeking a nectar reward and unlikely to differentiate among stamens, would not promote the evolution of heterantherous flowers and that bat‐pollinated flowers would be less likely than others to have differing stamen morphologies.

We used staminode descriptions for *Dillenia* species to examine their possible association with bat pollination. The roles of staminodes in flowering plants are many; they may be associated with nectar production, holding, cover or visual cues, for example (Endress & Matthews, 2006). They can also filter insects for pollination (Pontes et al., 2022; Wang et al., 2019). The fate of these stamens that have lost the function of pollen production may depend on whether they assume new functions; their main function is the prevention of self‐pollination in magnoliid‐type flowers, although some also attract pollinators with signals and rewards (Walker‐Larsen & Harder, 2000). Increased pollination efficiency may result in stamen loss (Walker‐Larsen & Harder, 2000), but the role of staminodes in bat pollination is poorly known. Staminodes may produce volatiles attracting bats (Paiva et al., 2019).

Considering the significance of the genus *Dillenia* to evolutionary biology and conservation, and the difficulty to study some remote species in situ, our objectives were to (1) examine the morphology of *D. biflora* pollen and review the morphologies of other known *Dillenia* pollens, (2) review corolla behaviour and colour, and pollen presentation (anther openings, stamens and staminodes) for *Dillenia* species and (3) infer what these traits may mean for the ecology of bat pollination in the genus.

## 2 METHODS

### 2.1 Study species for pollen examination and site

**FIGURE 1:**
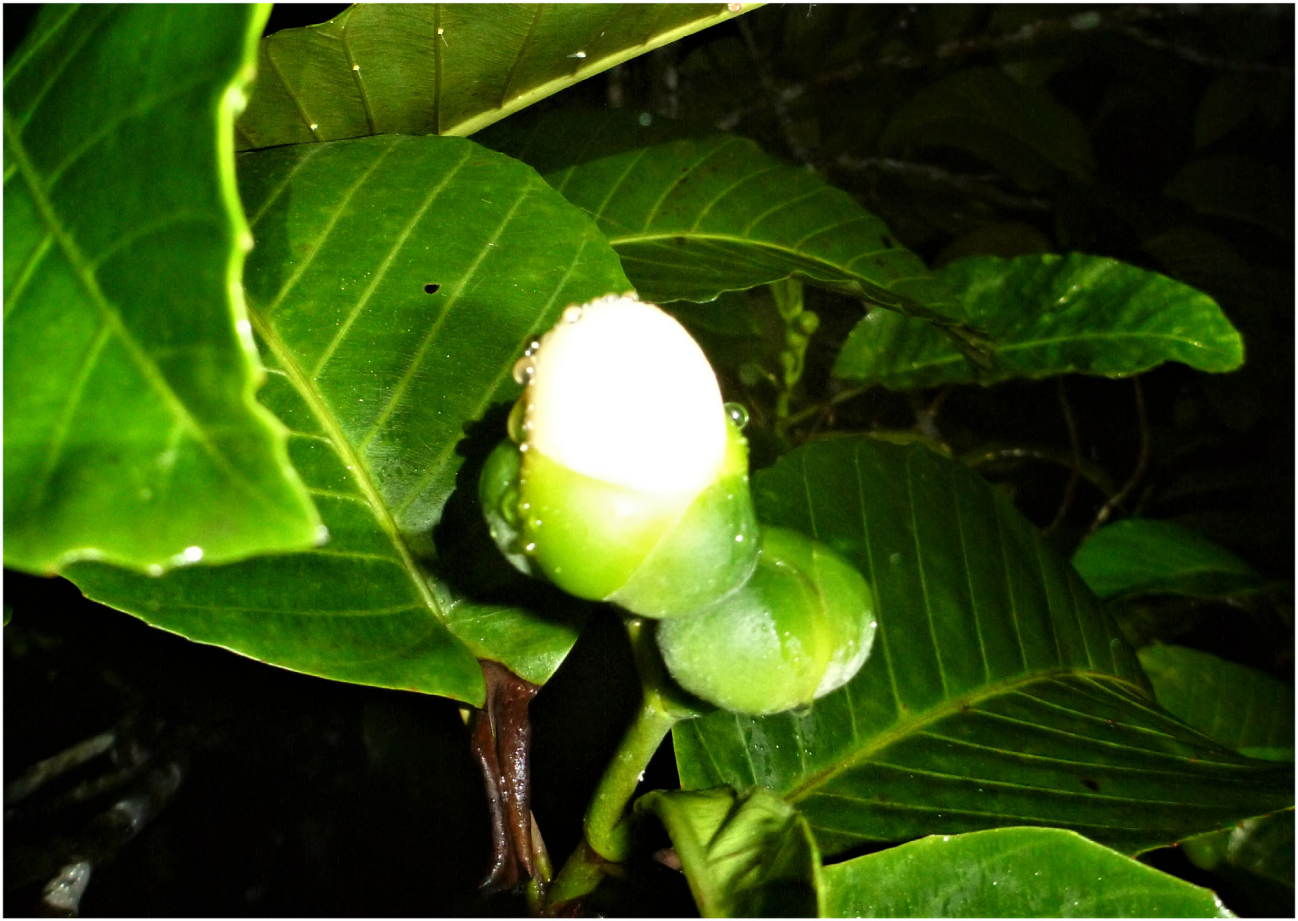
*Dillenia biflora* mature flower with permanently closed corolla until removal by a bat.

*Dillenia biflora* belongs to the class Dilleniales (eudicots) and occurs in primary and secondary forests and gardens in Fiji and Vanuatu. Known as kuluva in Fiji, it is an early coloniser used for timber and medicine, and it hosts threatened frogs (Scanlon, Petit, Tuiwawa, & Naikatini, 2014). The contents of its berries are likely consumed by bats and birds and consist of small black seeds attached to translucent white and fleshy funicular arils. The non‐seasonal cream to yellow and pink flowers have permanently closed globose corollas (16–20 mm measurements on average; Figure 1) that must be removed by bats with their teeth for pollination to take place. They contain nectar and some are lightly scented (Petit et al., 2022). Seeds are available for dispersal when the sepals curve back as the fruit opens upon maturity (Smith, 1981). Flowers are mature for only one night (Petit et al., 2022) and maturity is indicated by a fully globose and fresh corolla.

We reached the canopy of *D. biflora* trees using a ladder at Colo‐i‐Suva Rainforest Reserve (18°03′44″ S, 178°27′50″ E), Viti Levu, Fiji. Site access was granted by the Ministry of Forestry. In this tropical lowland rainforest, *D. biflora* is considered a sub‐canopy tree. The canopy is dominated by native trees such as *Myrsitica* spp., *Calophyllum* spp., *Garcinia* spp., *Endospermum macrophyllum*, and the introduced *Swietenia macrophylla* (mahogany). Among the native regrowth, mahogany stands planted in the 1940s and 1950s dominate all others in height, size and abundance. The total annual rainfall at Laucala Bay, ~9 km from the site, was 2891.2 mm in 2016 and the mean monthly temperature was 23.7°C (Fiji Meteorological Service, 2017).

### 2.2 Pollen analysis of *Dillenia biflora*

Pollen from three mature virgin flowers on different trees (DB 5.2, DB 6.8, DB19.5) was collected at night in December 2016 with the paper stem of a cotton applicator moistened with distilled water (Petit, 2011). It was kept in vials after drying at room temperature until mounting in June 2017 on microscope slides using fuchsin glycerine jelly (Beattie, 1971). It was inspected, measured and photographed with a Nikon eclipse Ci‐POL microscope (Tokyo, Japan) equipped with a Nikon DS‐Fi2 camera. We counted at least 150 pollen grains of each of the three flowers in at least three fields of view (100×). We counted only grains that presented a clear polar view of the colpi. We did not examine the details of the colpi by electron microscopy and use the general term colpus to describe the furrows in the grains (see Halbritter et al., 2018 for more detailed terminology).

### 2.3 *Dillenia* spp. flower attributes and bat ranges

A literature search for *Dillenia* and Dilleniaceae pollination and pollen (Google Scholar, Web of Science and Google) revealed a paucity of published research in the English language. We examined the monographs on *Dillenia* by Hoogland (1952, 1959) to determine corolla behaviour and pollen presentation for the *Dillenia* species. The unusual behaviour of some corollas is described by Hoogland (1952, 1959) as ‘falling as a whole without spreading’ or ‘petals falling off without spreading’, as is the case for *D. biflora*, which we know is pollinated by bats. Closed corollas requiring opening by bats would be grabbed as a whole and discarded on the forest floor, as with *D. biflora*, suggesting that this corolla behaviour may be associated with bat pollination. Other corollas were recorded as apetalous, had unknown behaviour, or were not specifically mentioned and we presumed they opened (‘the corolla [of Dillenia] usually consists of 5 free petals, which fall off within half a day after the flower has opened’ Hoogland (1952, p. 12)). We added to the list of species *D. tirupatiensis*, which was treated by Swamy et al. (2020) similarly to species in Hoogland (1952). We recorded the following traits: (1) ‘corolla falling as a whole without spreading’, unknown, or not noted as being either of those two options by Hoogland (1952, 1959), and for simplicity noted here as ‘corolla opening’, (2) petal colour, (3) presence or absence of staminodes, (4) stamen and staminode arrangement (categorised as stamens about the same length, stamens in two groups, stamens longer in the centre, and stamens shorter in the centre) and (5) position of the anther pore or slit. Regarding petal colours, although all but one were recorded by the same person (Hoogland, 1952, 1959), some of the specimens were dry and colour may have been recorded by other collectors in the field notes, so it is difficult to determine the accuracy with which colour was described. Descriptions were not given for all species (unknown or missing), and we do not know the number of specimens examined. Some qualifications (e.g., ‘deep cream’) could be interpreted differently. So, we categorised the flowers as yellow, white and cream or mixed within a species to determine whether one colour type was associated with ‘corolla falling as a whole’.

We searched the literature (Ali, 2022; Aninta et al., 2022; Bergmans & Rozendaal, 1988; Heaney, 1991; Irwin, 2017; IUCN Red List of Threatened Species, 2023; Kitchener et al., 1990; Koopman, 1982, 1989; Kruskop, 2011; Maryanto et al., 2012, 2019; Mickleburgh et al., 1992; Rozendaal, 1984; Sheherazade & Tsang, 2015; Tsang, 2015; Wiantoro, 2016; Zhang et al., 2010; local species lists; List of Mammals of the Philippines, 2023; iNaturalist Bangka‐Belitung, ID, 2023) to determine whether at least one bat species in the Pteropodidae (flying‐foxes and Old World blossom bats) occurred in the range of the *Dillenia* records. We did not attempt to determine the current exact distribution of each bat species because this information is unavailable. Similarly, the current distribution of each *Dillenia* species is poorly known.

### 2.4 Statistical analyses

We compared the frequency of staminode (vs. no staminode – not mentioned) and apical pore (vs. other anther openings, including pore near apex) observations between the groups with ‘corolla falling as a whole’ and ‘corolla opening’ with Fisher's exact test (IBM Corp., 2021). We used the same test to compare the frequency of stamens about the same length, stamens in two groups and stamens longer in the centre (omitting stamens shorter in the centre, represented by only one species) between species with ‘corolla falling as a whole’ and ‘corolla opening’. We compared similarly colour frequencies (yellow, white/cream and mixed) between the two groups.

## 3 RESULTS

### 3.1 Pollen heteromorphy

**FIGURE 2:**
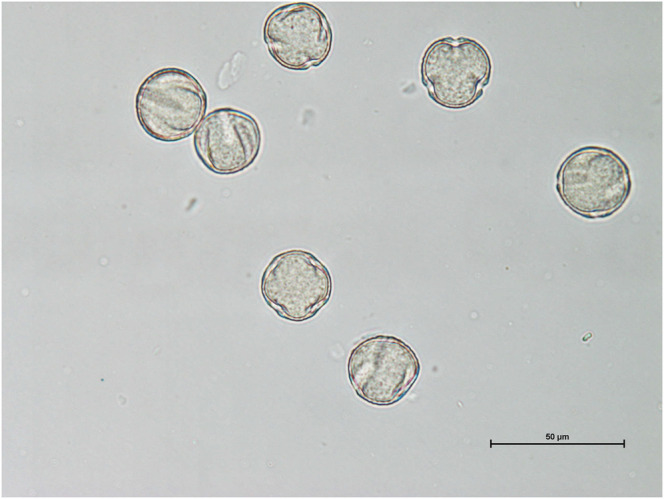
*Dillenia biflora* pollen grains from the same flower, showing three and four colpi.

The three virgin *D. biflora* flowers from different trees each contained both tricolpate and tetracolpate spherical pollen grains (25 μm diameter, with some shrinkage; Figure 2). We counted 148 tricolpate grains and 17 tetracolpate grains for DB 5.2 (8.7:1), 86 tricolpate and 126 tetracolpate for DB 6.8 (1:1.5), and 117 tricolpate and 62 tetracolpate for DB 19.5 (1.9:1). The literature search showed few studies on *Dillenia* pollen; they were not specifically looking for heteromorphy. Aperture heteromorphy (assumed, since it was not clear whether the samples were from the same individuals) occurred in a few species: *D. suffruticosa* (tri‐ to tetracolpate; Dickison, 1967; Smisha et al., 2016), *D. ovata* (tri‐ to tetracolpate; Dickison, 1967; Rakarcha et al., 2018), *D. luzoniensis* (tri‐ to tetracolpate; Dickison, 1967), *D. phillipensis* (tri‐ to tetracolpate; Rakarcha et al., 2018) and *D. castaneifolia* (dicolpate, tricolpate; ANU, 2023). *Dillenia andamanica* was noted as triporate, but only six grains were examined (Ganga Kailas et al., 2017).

### 3.2 *Dillenia* spp. flower attributes and bat ranges

We present data for 59 *Dillenia* species (Table 1 and Appendix 1). In addition to *D. biflora*, the species with ‘corolla falling as a whole’ are *D. schlechteri*, *D. papuana*, *D. crenata*, *D. salomonensis*, *D. insignis*, *D. pteropoda*, *D. nalagi*, *D. cyclopensis* and *D. quercifolia*. Of seven species with unknown corolla opening (*D. montana*, *D. ingens*, *D. talaudensis*, *D. marsupialis*, *D. fagifolia*, *D. blanchardii* and *D. insularum*), the first two have corollas that possibly fall as a whole according to Hoogland (1952, 1959), who noted (Hoogland, 1952) for *D. ingens* that ‘it is possible that the petals fall without spreading as e.g. in *D. papuana* […] as the present species is found in the area, where species with normally spreading corolla have not (yet?) been found’. For all the other species presented in Appendix 1, the behaviour of the corolla was not mentioned as unknown or possibly falling as a whole.

**TABLE 1:** Observations of staminodes, anther openings, stamen length and colour by Hoogland (1952, 1959) for 58 *Dillenia* species (see Appendix 1) plus *D. tirupatiensis* (Swamy et al., 2020).

	Corolla ‘falling as a whole’	Corolla behaviour unknown or apetalous	Corolla ‘opening’
Total number of species	10	7	42^a^
Species with staminodes	3	2	5
No staminode observation	7	5	37
Apical anther pore	9	4	8
Anther pore near apex	1	1	26
Mixed anther pores: apical and near apex	0	1	1
Anther with longitudinal slits	0	0	7
Anther opening unknown or not mentioned	0	1	0
Stamens about the same length	8	3	13
Stamens in two distinct groups	0	2	23
Stamens longer towards centre	2	1	5
Stamens shorter towards centre	0	1	1
Yellow colour	3	2	25
White and cream colour	2		11
Mixed colours	3		3

*Note*: Corolla ‘opening’ indicates that flowers are not listed as unknown or having ‘corolla falling as a whole’ by Hoogland (1952, 1959). Colour is interpreted and summarised by the authors (see Section 2).

^a^ 43 species are listed in Hoogland (1952, 1959), but two are lacking information about the androecium.

The petal colours of several *Dillenia* species were unknown or not mentioned. Three species with ‘corolla falling as a whole’ had yellow corollas, two had white or cream corollas and three had mixed colours. Hoogland (1952) indicated that both yellow and white had been written on the field labels for this species in Fiji and Vanuatu and that it was the only species in the genus in which both colours were found. However, he also recorded yellow or white for *D. crenata* from the Solomon Islands. In Fiji we observed *D. biflora* flowers that were cream to yellow and even pink. Among species with unknown corolla opening, two had yellow corollas. As for the species with ‘corolla opening’, 25 had yellow petals, 11 had white or cream petals and three had mixed colours (Table 1 and Appendix 1). Yellow was more common in the ‘corolla opening’ group than expected, whereas mixed colour was more common in the ‘corolla falling as a whole’ group (Fisher's exact test *p <* .001).

Three of 10 (30.0%) *Dillenia* species with ‘corolla falling as a whole’ had staminodes, as did two of seven (28.5%) species with unknown corolla behaviour and five of 42 (11.9%) species with ‘corolla opening’ (Fisher's exact test *p* = .171 for the comparison of staminode frequency between ‘corolla falling as a whole’ and ‘corolla opening’; Table 1 and Appendix 1). Two of the latter (total = 44 species) are not counted here because some characteristics were not mentioned (from Hoogland, 1952, 1959).

Eight of 10 trees (80%) with ‘corolla falling as a whole’ had stamens all about the same length except for two that had longer stamens towards the centre, including *D. biflora*. Three out of seven (42.9%) with unknown corolla behaviour had stamens about the same length (including the two suspected of having ‘corolla falling as a whole’), two had stamens in two distinct groups (28.6%), while one (14.3%) had stamens that increased in size towards the centre and another's stamens decreased in size towards the centre. Among trees with ‘corolla opening’, 13 of 42 (31.0%) had stamens of about the same length; the stamens of five (11.9%) increased in size towards the centre and those of 23 (54.8%) were in two distinct groups, whereas the stamens of one species (2.4%) decreased in size towards the centre. Stamens in two distinct groups and shorter towards the centre did not occur among species with ‘corolla falling as a whole’ (Table 1). Species with ‘corolla falling as a whole’ were proportionately more likely to have stamens about the same length than did species with ‘corolla opening’, and we would have expected over four of 10 species to have stamens in two groups, but none did (Fisher's exact test *p* < .001).

Nine of 10 *Dillenia* species (90%) with ‘corolla falling as a whole’ had apical anther openings and one with the opening near the apex. For species with unknown corolla behaviour, four out of seven (57.1%) had apical pores, including the two suspecting of having ‘corolla falling as a whole’, one was mixed, one had pores near the apex and one was unknown. For species with ‘corolla opening’, eight of 42 (19.0%) had apical pores, 26 (61.9%) had pores near the apex, one (2.4%) was mixed and seven (16.7%) had longitudinal slits (Fisher's exact test *p* < .001 for apical pores vs. others between ‘corolla falling as a whole’ and ‘corolla opening’).

At least one bat species in the family Pteropodidae occurred in the general range of each *Dillenia* species presented in Hoogland (1952, 1959). Plant ranges are summarised in Appendix 1.

## 4 DISCUSSION

### 4.1 Pollen heteromorphy

The pollen samples from three different plants showed great diversity in heteromorphy for *D. biflora*. One had 1.5 times more tetracolpate than tricolpate grains, whereas tricolpate grains dominated the other two, one by nearly double the number and the other nearly nine times the number of tetracolpate grains. The fact that heteromorphy (or possibly polymorphism – not specified) was observed in India for *D. suffruticosa*, a nectarless plant pollinated principally by bees (Smisha et al., 2016, as it is also in Malaysia, Tokumoto et al., 2014) indicates that this character is not reserved to bat‐pollinated plants. The size of the grains was similar to those of *D. biflora* (25 μm) and anther dehiscence started 16–17 h earlier than did anthesis, making the one‐day flowers protandrous (Smisha et al., 2016). Pollen contained protein but no starch or lipids, and its viability declined steadily by the end of the day of anthesis (Smisha et al., 2016). The Australasian pollen and spore atlas (ANU, 2023) presents photographs of a few pollen grains of *D. castaneifolia* (dicolpate, tricolpate) from Papua New Guinea, with corolla that presumably opens. In Thailand, Rakarcha et al. (2018) found tricolpate and tetracolpate pollen in *D. ovata* and the cultivated *D. philippensis*. The 11 other *Dillenia* species were classified as tricolpate, including *D. suffruticosa*. These findings indicate that pollen morphology is poorly known among *Dillenia*, and that heteromorphy is not reserved to plants likely to be pollinated by bats. However, the large differences in pollen aperture ratios among our three *D. biflora* flowers are remarkable and not observed in other species, for which the tetracolpate form was ‘rarely or occasionally’ encountered in samples (Dickison, 1967).

Pollen heteromorphy is relatively common (e.g., references in Dajoz et al., 1991 and Harley, 1991; Pire & Dematteis, 2007), so our findings for *D. biflora* are not unexpected. For example, Harley (1991) noted that pollen morphology in the Sapotaceae could differ even within species. In this family, the pollens of most genera did not have a consistent number of apertures, with most being 3–4 or 4–5 colporate. Interestingly, many Sapotaceae are pollinated or likely to be pollinated by bats (Aziz et al., 2021; Harley, 1991), including in Fiji (Scanlon, Petit, Tuiwawa, & Naikatini, 2014), as are some species in the Sapindales (Fleming et al., 2009), a group known to have genera with heteromorphic pollen (Gonçalves‐Esteves et al., 2022). Harley (1991) found that the ratio within a sample of one aperture number to the other was generally 0.25–0.30 to 0.65–0.75. The percentage of three‐apertured pollen was around 30% (for three and four total) in *Viola diversifolia* (Dajoz et al., 1991), while for ours it was 41%, 65% and 90%. It is unclear how these ratios occur, although pollen morphogenesis is receiving much attention (e.g., Albert et al., 2022; Matamoro‐Vidal et al., 2016). What could be ecological advantages? Dajoz et al. (1991) proposed that the faster germination of violet pollen with four apertures, but the faster tube growth and greater survival of pollen with three apertures, favoured the coexistence of both morphologies. Božič and Šiber (2020) demonstrated that as the number of pollen apertures increases, the necessary condition of appropriate softness for successful infolding during desiccation becomes more stringent. Although a large number of apertures maximises the likelihood that one will contact the stigma, with the potential added benefits of fast germination (Dajoz et al., 1991), they may also render pollen more sensitive to osmotic stress (Prieu et al., 2016). Božič and Šiber (2020) suggested that the prevalence of one or three apertures in the angiosperms could be due to a robust mechanical design allowing tidy infolding and closure of the grains against desiccation. Pollen undergoes some dehydration before dispersal, and environmental conditions affect its behaviour (Franchi et al., 2011). We would expect that long‐distance pollen dispersal by bats could result in some desiccation, against which pollen must be protected, and a resulting mix of three‐ and four‐aperturate pollen grains. Steady‐state flowering plants with few mature flowers per plant in both wet and dry seasons, such as *D. biflora*, with short‐lived flowers in humid environments and with fast long‐distance pollinators, should benefit from heteromorphic pollen. It would be interesting to determine whether *D. biflora* populations in different climatic zones and at different altitudes would present different aperture ratios, and whether other presumed chiropteropisunous *Dillenia* species would have similar characteristics. Although our discussion focuses on bat pollinators, the same comments could apply to other pollinators, and it is possible that pollen heteromorphy suits many types of pollination in tropical environments. Intraspecific pollen morphology needs more attention if we are to understand its ecological significance and evolution.

Certainly, the closed corolla of *D. biflora* before pollen dispersal would not only prevent nectar dilution by rain (Petit et al., 2022) and improve the longevity of pollen (Hase et al., 2006; Mao & Huang, 2009; Pacini & Franchi, 2020), but also protect pollen from precocious hydration and germination within anthers or misplaced germination, which could be disastrous if hydration was not regulated (Edlund et al., 2004). Nectar spillage is common in some bat‐pollinated flowers (Domingos‐Melo et al., 2021) and a closed corolla could also prevent spillage, although it is unlikely to be a problem for the sturdy sepal cups of *D. biflora*. We may additionally envisage that the closed corolla could facilitate nectar reabsorption by unvisited flowers, as occurs in some bat‐pollinated species (e.g., Avila Jr et al., 2022), or allow a combination of all these options. However, the protection of nectar and pollen in high‐rainfall environments, as well as selection of the best pollinators, are great advantages of a chiropterosunous plant with closed corolla.

### 4.2 Corolla colour

Flower colour is a key element of angiosperm diversity that affects pollinator attraction and may also have a role in stress resistance (Narbona et al., 2021). Colour plasticity may be influenced not only by pollinators but also by herbivores (Sobral et al., 2021). Many factors affect the optical properties of flowers for signalling (van der Kooi et al., 2019). Colour interpretation, which depends on the sensory capabilities of the visitors, may be modified by context (Garcia et al., 2020). Flower perception is also affected by correlated traits, abiotic factors (Narbona et al., 2021), and other cues such as acoustics and scent (Gonzalez‐Terrazas et al., 2016; Raghuram et al., 2009), making our understanding of colour choice by bats difficult. Nevertheless, the colour polymorphism of many *Dillenia* species makes it a promising field of study to improve our understanding of the evolution of pollination systems in this genus. Our findings on *Dillenia* petal colours do not currently permit us to allocate specific colours to different corolla behaviours since different colours occur in each category, but flowers with ‘corolla falling as a whole’ were more likely than flowers with ‘corolla opening’ to have mixed colours and less likely to be yellow. Only 7.7% of species with ‘corolla opening’ had mixed colours, whereas 37.5% of species with ‘corolla falling’ as a whole did, including the bat‐pollinated *D. biflora*, recorded by Hoogland (1952) as having white and yellow flowers, and by Petit et al. (2022) also to have pink ones. White, cream and yellow certainly offer to bats a strong contrast against dark backgrounds in the rainforest, whether it is by appearing chromatic during twilight or bright at night (see Domingos‐Melo et al., 2021). A variety of colours may indicate a shift away from bee pollination. Interestingly, red flowers occur among mixed colours in *D. pteropoda* (‘corolla falling as a whole’) and two other species in the ‘corolla opening’ group. The ‘apricot to dark pink’ flowers were recorded only for *D. insignis*. The role of red, dark pink and apricot in pollinator attraction should be investigated (see Lunau et al., 2011). The study of *Dillenia* flower colour, UV reflectance (e.g., Domingos‐Melo et al., 2021) and colour saturation perception by bees and different vertebrates (e.g., Lunau et al., 2011) would most likely help to understand the evolution of pollinating systems in the Dilleniaceae. Colour may act in concert with scent (Gonzalez‐Terrazas et al., 2016), and the delicate scent of some *D. biflora* flowers suggests that the analysis of floral volatiles could contribute to the elucidation of pollination in this genus.

### 4.3 Stamens and staminodes

In his examination of several *Dillenia* species, Endress (1997), like Hoogland (1952, 1959), found that some were heterantherous. In the heterantherous *D. indica*, the corolla opens and stamens are in two groups (Rizzo et al., 2020). Although the central anthers contained spherical pollen, the peripheral ones also contained pollen of other irregular shapes in addition to spherical grains (oval, pentagonal, square and other irregular shapes; Rizzo et al., 2020), and it was suggested that this pollen could be ‘feed’ for pollinators (as discussed for other genera by Anderson & Symon, 1989 and Lloyd, 2000). In our study, the striking difference between the species suspected to be pollinated by bats and the ones with corollas that are thought to open was the prevalence (54.8%) of species with stamens in two distinct groups for ‘corolla opening’, a characteristic not seen among the species with ‘corolla falling as a whole’. This finding supports that of Vallejo‐Marín et al. (2010), suggesting that heteranthery is associated with nectarless plants (families in their study) mostly pollinated by bees. Bats are unlikely to discriminate among anther and stamen morphs, and bat pollination may promote stamen uniformity. The androecia of many species of Melastomataceae have dimorphic anthers, a feature that may enhance attractiveness to bees and facilitate their grasping of the anthers (Renner, 1989). Intermediate levels of heteranthery and stylar dimporphism resulted from bee‐mediated selection in *Macairea radula* (Melastomataceae) (Oliveira et al., 2020). Stamen dimorphism is more likely to evolve when pollinators can discriminate between morphs offering different rewards, as described by Dellinger, Artuso, et al. (2021) for bird visitation of Melastomaceae, which allows for staggered pollen release in some species. (Interestingly, the Melastomaceae has many evolutionary and ecological points in common with Dilleniaceae. Renner (1989) stated that ‘in nectariferous melastomes, the petals do not spread at anthesis but remain convolute almost like in buds’). Functions of heteranthery may vary in a non‐mutually exclusive way (Dellinger, Artuso, et al., 2021), but for chiropteropistusis, anthers simply need to maximise contact with bats, particularly the part most likely to contact the stigma of the next flower and improve flower visibility once the corolla has been removed to encourage repeated visitation (secondary function of staminodes, Walker‐Larsen & Harder, 2000). Our results suggest that stamens all about the same length or slightly longer in the centre of the flower, as present in *D. biflora*, should represent the best structure for bat pollination, possibly by maximising contact with the bat's muzzle. Among species with ‘corollas falling as a whole’ (Appendix 1) outer staminodes could represent a shift from bee pollination and/or have functions that promote the detection of flowers once corollas have been removed. Based on our findings, it is reasonable to expect that among the seven species in Appendix 1 with unknown corolla behaviour, at least the two from Papua New Guinea and the Solomon Islands suspected of having ‘corollas falling as a whole’ by Hoogland (1952, 1959), and which have apical pores and stamens about the same length, could be bat‐pollinated. Only field research can verify this hypothesis.

### 4.4 Pollen presentation in the anther

The position of anther pores affects the directionality of pollen release. For example, anther pore and rostrum in *Rhynchanthera grandiflora* (Melastomataceae) focus the pollen ejected by buzz pollination on bees, and two stamen lengths improved the coverage of the bees' bodies with pollen (Konzmann et al., 2020). In *D. biflora*, the anthers of the outermost stamens tended to be shorter than the innermost ones, sometimes with a single theca; the linear thecae opened with apical pores (Hoogland, 1952), as in nine out of 10 species with ‘corollas falling as a whole’ (Table 1). The frequency of species with apical pores as opposed to other openings was significantly greater in this group than in the group with ‘corolla opening’, which had a large proportion of species with anthers with pores near the apex. The subtle difference of pollen release between pores at the apex and near the apex is mysterious when it comes to bats. It is likely that pollen release via an apical pore is more effective for bat pollination, as the salt‐shaker pollen expulsion mechanism reported by Dellinger, Chartier, et al. (2019) and Dellinger, Pérez‐Barrales, et al. (2021). Pollen presentation may suit different pollination styles. For example, perching bats are more effective pollinators than are hovering bats in the New World palm *Calyptrogyne ghiesbreghtiana* (Tschapka, 2003). The feeding behaviour of bats at *D. biflora* presented in Petit et al. (2022, 2021), which involves perching and approaching the flower from the top with the tip of the muzzle, is obviously compatible with pollen dispensing via apical pores.

## 5 CONCLUSIONS

Flower and anther closure protect pollen from humidity and rain during presentation (Pacini & Franchi, 2020), conferring an advantage to plants keeping their corollas closed until pollination in high‐rainfall environments. *Dillenia* flowers have many parts (Endress, 1997; Hoogland, 1952) and floral modularity facilitates evolvability via pollinator‐mediated selection (Dellinger, Artuso, et al., 2019). Like certain tribes of the Melastomataceae, *Dillenia*'s intraspecific pollen morphological diversity (Dickison et al., 1982), including different numbers of apertures (Rakarcha et al., 2018; this study) may facilitate pollen dispersal by bats in tropical environments. *Dillenia biflora* pollen can be dispersed in wet or dry conditions on the fur of bats in the Fijian rainforest; bats can travel long distances, suggesting that pollen grains would be subjected to dramatic changes in conditions. Aperture heteromorphy in this species may represent a trade‐off between germination speed and survival rate as in *A. thaliana* (Prieu et al., 2016), but the effect of colpi is currently speculative for *D. biflora*, and its pollen properties (e.g., elasticity) could be very different to those of previously studied species. We recommend the study of pollen morphology in more *Dillenia* species, including with electron microscopy. Different populations of *Dillenia* species could feature different morphologies (intra‐ and inter‐specific) depending on environmental conditions, making the genus an exciting model to test hypotheses.

Anther alteration can facilitate the utilisation of novel pollination environments (Kemp & Vallejo‐Marín, 2021). Apical anther pores are strongly associated with ‘corollas that fall as a whole’ in *Dillenia*, and probably represent an effective way of delivering pollen to bats; it would be interesting to compare the trajectory of the pollen released from apical pores with that of pollen expulsed from pores near the apex. Similarly, *Dillenia* stamen dimorphism is not expected to be selected by strong evolutionary pressure resulting from bat pollination, apart from possibly longer stamens in the centre of the flower, as occurs in *D. biflora*. If chiropteropisteusis is confirmed, staminodes on some species may be remnants of ancestral bee pollination and/or enhance perception by visitors once the corollas are removed. Several other characters such as large flower size (*Xylocopa*‐like bee‐pollinated flowers may facilitate shifts to vertebrate pollination; Arroyo, 1981), petal colour (for which a shift may be indicated by mixed colours within species in *Dillenia* and a reduction in yellow morphs), scent and most importantly the presence of nectar, only established for *D. biflora* (Petit et al., 2022), need further examination to confirm suitability for, or relationship with, bat visitation.

Dellinger et al. (2022) estimated a shift to nectarivorous vertebrate pollination in 146 (2.5%) Melastomataceae species, a family mostly buzz‐pollinated. Shifts from bee to vertebrate pollination may have similarly taken place for several species in the Dilleniaceae. We found that the range of all *Dillenia* species in Appendix 1 matches that of bat species in the Pteropodidae. Chiropterophily is not an evolutionary dead end (Oliveira et al., 2021), but chiropteropisteusis could be for some *Dillenia* species. The total dependence of chiropteropisunous trees on bats and strong dependence of bats on those trees, as is the case in Fiji, could endanger the participants in several mutualisms considering the threatened status of many island bats and some *Dillenia* tree species (Petit et al., 2022).

## AUTHOR CONTRIBUTIONS

**Sophie Petit:** Conceptualization (lead); data curation (lead); formal analysis (lead); funding acquisition (lead); investigation (lead); methodology (lead); project administration (lead); writing – original draft (lead). **Annette T. Scanlon:** Funding acquisition (supporting); investigation (supporting); writing – review and editing (supporting). **Alivereti Naikatini:** Funding acquisition (supporting); investigation (supporting); writing – review and editing (supporting). **Tara Pukala:** Funding acquisition (supporting); investigation (supporting); writing – review and editing (supporting).

## CONFLICT OF INTEREST STATEMENT

The authors declare no conflict of interest.

## Data Availability

The data that support the findings of this study are available in the paper.

## APPENDIX 1

### 1.1

*Dillenia* species likely to be pollinated by bats based on description by Hoogland (1952 – indicated as ^1952^, 1959 – indicated as ^1959^) of petals ‘falling as a whole without spreading’ (not shaded), with corolla behaviour unknown (light grey) and with corollas not indicated to be unknown or possibly falling as a whole by Hoogland (dark grey). *Dillenia tirupatiensis* is from Swamy et al. (2020).

**Table:**

Species	Hoogland (1952) Species number	Hoogland (1959) Species number	Geographic distribution as described by Hoogland (1952, 1959)	Corolla ‘falling as a whole without spreading’ (X) or unknown (U)	Petal colour as described by Hoogland (1952, 1959)	Anther pore or slit	Staminodes present according to Hoogland (1952, 1959)	Stamens, staminodes
*D. schlechteri*	10		E [Papua] New Guinea and New Ireland [PNG]	X	Bright yellow	Apical	No	Stamens all about same length
*D. papuana*	13		Lesser Sunda [Nusa Tenggara] Islands (Tanimbar Islands only) [Indonesia], New Guinea and adjacent islands (some islands in the Geelvink Bay and Aru Islands [Indonesia])	X	Yellow	Apical	No	Stamens all about same length
*D. crenata*	14		Solomon Islands	X	Yellow or white	Apical	No	Stamens all about same length
*D. salomonensis*	15		Solomon Islands	X	Deep cream	Apical	No	Stamens all about same length
*D. insignis*	16		Solomon Islands	X	Apricot to dark pink	Apical	No	Stamens all about same length
*D. biflora*	17		Fiji Islands and S New Hebrides [Vanuatu]	X	Yellow or white	Apical	No	Slightly longer innermost stamens
*D. pteropoda*	6	1	Moluccas [Maluku Islands, Indonesia] (Halmaheira [Halmaheira], Batjan [Bacan]), Salawati [SW Papua, Indonesia]	X	White^1952^, red	Near apex on outer side^1952^	Yes^1952^	Stamens all about same length; few staminodes on outer side of androecium^1952^
*D. nalagi*		4	Papua (Buna‐Kokoda area, Northern District) [PNG]	X	Yellow	Apical	Yes	Stamen length increases towards centre; staminodes on the outside
*D. cyclopensis*		7	Cyclops Mountains [Indonesia]	X	NM	Apical	Yes	Stamens all about same length; about 10 staminodes on the outside
*D. quercifolia*	9	5	SE New Guinea (Northern and Milne Bay Districts [Oro and Milne Bay Provinces]), Fergusson Island^1959^ [PNG]	X^1959^	Unknown	Apical	No	Stamens all about same length
*D. montana*	11	3	NE New Guinea (Central Highlands and Hunstein Range) [PNG]	U (possibly falling as a whole^1959^)	Yellow	Apical	No	Stamens all about same length
*D. ingens*	12		Solomon Islands	U (possibly falling as a whole because species with ‘normal’ corollas not found in the area)	Unknown	Apical	No	Stamens all about same length
*D. talaudensis*	24		Moluccas [Maluku Islands, Indonesia], only Salibabu Island	U	Unknown	Apical for outer group, near apex on outer side for inner group	No	Stamens in 2 distinct groups
*D. marsupialis*	28		Philippines (Luzon, Catanduanes and Panay only)	U	Unknown	Near apex on outer side	No	Stamens in 2 distinct groups
*D. fagifolia*	31		New Guinea near Aitape [PNG]	U	Unknown	Unknown	Yes	Unique androecium in genus; stamens decrease in size towards centre; staminodes within stamens
*D. blanchardii*	40		Indo‐China [Vietnam], only Annam and Poulo Condor [Côn Sơn]	U	Yellow	Apical	No	Stamens of different sizes, increasing towards centre
*D. insularum*		6	Sudest [Vanatinai or Tagula] Island (‘primary relic rain forest’), Misima Island [PNG]	U	Unknown	Apical	Yes	Stamens 6–10 mm long; staminodes on the outside
*D. beccariana*	1		Borneo, Sarawak only [Malaysia]		Yellow	Near apex on outer side	No	Stamens all about same length (outermost ones slightly shorter)
*D. albiflos*	2		Malay Peninsula, SE Johore only [Malaysia]		Pale cream white	Near apex on outer side	No	Stamens all about same length (outermost ones slightly shorter)
*D. celebica*	3		Celebes [Sulawesi, Indonesia], only eastern part of North Peninsula and Lake Towuti region		Apetalous	Longitudinal lateral slit	No	Stamens all about same length
*D. serrata*	4		Celebes [Sulawesi, Indonesia]		Apetalous	Apical	No	Stamens all about same length (inner ones slightly longer)
*D. fischeri*	5		Philippines, only Mindanao		White	Near apex on outer side	No	Stamens all about same length (decreasing in size towards centre)
*D. triquetra*	7		eastern Madagascar, Ceylon [Sri Lanka]		White	Near apex on outer side	No	Stamens all about same length
*D. ovifolia*	8		Japen [Yapen] Island, Moluccas [Maluku Islands] (Halmaheira [Halmahera] and Morotai) [Indonesia]		White	Apical	No	Stamens all about same length
*D. ferruginea*	18		Seychelles Islands		Whitish	Longitudinal lateral slit	Yes	Stamens and staminodes together; appear to be longer towards the centre
*D. alata*	19		Waigeo Island, S New Guinea [Indonesia], and Queensland [Australia]		Yellow	Near apex on inner side	No	Stamens in 2 distinct groups
*D. auriculata*	20		New Guinea [Indonesia and PNG]		Yellow	Apical for outer group, near apex on outer side for inner group	No	Stamens in 2 distinct groups
*D. castaneifolia*	21		New Guinea [PNG and Indonesia] and some islands in Geelvink Bay (Japen [Yapen], Korido [Supiori?]) [Indonesia]		Deep lemon yellow	Near apex on inner side for outer group, near apex on outer side for inner group	Yes	Stamens in 2 distinct groups, often staminodes on the outside
*D. bolsteri*	22		Philippines; Samar, Leyte and Surigao Province of Mindanao only		White	Near apex on inner side	No	Stamens in 2 distinct groups
*D. diantha*	23		Philippines, Luzon only		Yellow	Near apex on outer side	No	Stamens in 2 distinct groups
*D. ochreata*	25		Celebes [Sulawesi, Indonesia], Minahasa only		Yellow	Apical	No	Stamens in 2 groups
*D. megalantha*	26		Philippines; Luzon, Samar, and Mindanao		Yellow	Near apex on inner side for outer group, near apex on outer side for inner group	No	Stamens in 2 distinct groups
*D. philippensis*	27		Philippines, most islands from the Babuyan Islands to the Sulu Islands (not in Palawan and the Calamianes); var. *pubifolia* known only from Luzon and Mindanao		White	Apical	No	Stamens in 2 distinct groups
*D. reifferscheidia*	29		Philippines, from Luzon to Mindanao		White, rose red	Near apex on outer side	No	Stamens in 2 distinct groups
*D. suffruticosa*	30		Sumatra (only Palembang), Malay Peninsula, Riouw [Riau] and Lingga Archipelagos [Indonesia], Banka [Bankga Island, Indonesia], Billiton [Belitung], Borneo [Malaysia, Brunei, Indonesia], W Java [Indonesia], Philippines [suspected naturalised there]		Bright yellow	Near apex on outer side	Yes	Stamens getting longer towards the inside; staminodes on outer side
*D. exemia*	32		Sumatra [Indonesia], Malay Peninsula and Borneo [Malaysia, Brunei, Indonesia]		Apetalous	Apical	No	Stamens all about same length
*D. reticulata*	33		Sumatra [Indonesia], Malay Peninsula and Borneo [Malaysia, Brunei, Indonesia]		Yellow	Apical	No	Stamens increasing in size towards centre
*D. borneensis*	34		Borneo [Malaysia, Brunei, Indonesia]		Yellow	Longitudinal lateral slit	No	Stamens decreasing in size towards centre
*D. mansoni*	35		Burma [Myanmar]and possibly Assam [India]		White	Near apex on outer side	No	Stamens all about same length
*D. bracteata*	36		Deccan Peninsula (Nilgiris on W coast and Kambakan Hill [?] near Madras [Chennai] on E coast); Ceylon [Sri Lanka] record probably an error		Yellow	Near apex on outer side	No	Stamens all about same length
*D. hookeri*	37		Siam [Thailand], S to Satul and Indo‐China [Vietnam], N to ca. 17° N		Yellow	Apical	No	Stamens all about same length
*D. excelsa*	38		Sumatra, Malay Peninsula and Borneo [Malaysia, Brunei, Indonesia]; less common in W Java; one record of var. pubescens from Balabac Island (Philippines)		Bright yellow	Near apex on inner side	No	Stamens in 2 distinct groups
*D. luzoniensis*	39		Philippines, only Palawan and W Luzon [seemed to have disappeared from Luzon]		Yellow	Near apex on inner side	Yes	Stamens in 2 distinct groups; often some staminodes outside outer stamens
*D. sumatrana*	41		Sumatra and some adjacent islands (Pulau Bras [? not near Sumatra but N of W Papua; may be an error], Simalur [Simeulue?], Nias, and Siberut) [Indonesia], Malay Peninsula (not in the southernmost part) and Borneo (only Sarawak and British North Borneo [North Borneo]) [Malaysia]		Yellow	Near apex on outer side	No	Stamens in 2 distinct groups
*D. sibuyanensis*	42		Sibuyan Island only (Philippines)		Lemon yellow	Near apex on outer side	No	Stamens in 2 distinct groups
*D. retusa*	43		Ceylon [Sri Lanka]		White	Near apex on outer side	No	Stamens increasing in size towards centre
*D. monantha*	44		only Palawan and the Calamianes [Calamian Islands] (Philippines)		Yellow	Near apex on outer side	No	Stamens increasing in size towards centre
*D. turbinata*	45		S China (Kwangsi [Guangxi] and Hainan) and Indo‐China [Vietnam] (Tonkin and Annam), S to ca. 15° N		Bright yellow, white, pink	Near apex on outer side	No	Stamens in 2 distinct groups
*D. ovata*	46		Siam [Thailand] and Indo‐China [Vietnam] N to about 17°30′ N latitude, Sumatra [Indonesia], Malay Peninsula [representing three countries] (not in Johore and Singapore) and Banka [Bankga Island, Indonesia]		Lemon yellow	Near apex on outer side	No	Stamens in 2 distinct groups
*D. indica*	47		India, Burma [Myanmar], S China (Yunnan), Siam [Thailand], Indo‐China [Vietnam], Sumatra [Indonesia], Malay Peninsula, Java [Indonesia] and Borneo [Indonesia]; [origin unknown (cultivated)]		White with green veins	Near apex on outer side	No	Stamens in 2 distinct groups
*D. pulchella*	48		Sumatra, Malay Peninsula, Riow [Riau]‐Lingga Archipelago [Indonesia], Banka [Bankga Island, Indonesia] and Borneo [Malaysia, Brunei, Indonesia]		Yellow	Longitudinal lateral slit	No	Stamens in 2 distinct groups
*D. parkinsonii*	49		Burma [Myanmar] and possibly Assam [India]		Unknown	Apical	No	Stamens all about same length
*D. pentagyna*	50		India, Burma [Myanmar], Andaman Islands [India], China (Yunnnan, Hainan), Siam [Thailand], Indo‐China [Vietnam], Java (not westernmost part), Lesser Sunda Islands [Nusa Tenggara, Indonesia], S Celebes [Sulawesi]		Yellow	Extrorse with longitudinal slits	No	Stamens in 2 distinct groups
*D. scabrella*	51		Bengal [India], Assam [India], Burma [Myanmar] and Indo‐China [Vietnam], possibly Nepal		Bright yellow	With longitudinal slits	No	Stamens in 2 distinct groups
*D. parviflora*	52		Burma [Myanmar] and W Siam [Thailand]		Yellow	Longitudinal lateral slit	No	Stamens in 2 distinct groups
*D. andamanica*	53		Andaman and Nicobar Islands [India]		Yellow	Near apex on inner side	No	Stamens in 2 distinct groups
*D. aurea*	54		India (not Deccan Peninsula, Bengal and Assam), Burma [Myanmar], NE Siam [Thailand]		Yellow	Near apex on inner side for outer group, near apex on outer side for inner group	Yes	Stamens in 2 distinct groups; sometimes staminodes outside outer stamens
*D. obovata*	55		lower Burma [Myanmar], Siam [Thailand], Indo‐China [Vietnam], northern part of Malay Peninsula, S Sumatra and W Java (E to Tegal and Nusa Kambangan) [Indonesia]; Borneo record not reliable		Bright yellow	Near apex on inner side for outer group, near apex on outer side for inner group	Yes	Stamens in 2 distinct groups; sometimes staminodes outside outer stamens
*D. papyracea*		1	Philippines (Luzon, Basilan, Mindanao)		NM	NM	NM	NM
*D. ovalifolia*		2	Waigheo [Waigeo] and Sorong [SW Papua, Indonesia]		White [?], pink, red	NM	NM	NM
*D. tirupatiensis*			India (Chittoor District of Eastern Ghats, Andhra Pradesh)		White	Near apex	No	Stamens all about same length

*Note*: Words in brackets added by the authors. At least one Pteropodidae (fruit and blossom bats) bat species was present at all locations, from Koopman (1982), Rozendaal (1984), Bergmans and Rozendaal (1988), Koopman (1989), Kitchener et al. (1990); Heaney (1991), Mickleburgh et al. (1992), Zhang et al. (2010), Kruskop (2011), Maryanto et al. (2012), Sheherazade and Tsang (2015), Tsang (2015), Wiantoro (2016), Irwin (2017), Maryanto et al. (2019), Ali (2022), Aninta et al. (2022), IUCN Red List, local species lists, List of Mammals of the Philippines (2023), iNaturalist Bangka‐Belitung, ID (2023). Staminodes on outer side of androecium (except *D. fagifolia*, inner side). *D. pteropoda* was assumed to have ‘normal’ corolla in Hoogland (1952), but was revised in Hoogland (1959); the species is treated with *D. papyracea* in Hoogland (1959). *D. nalagi* resembles *D. biflora*, but has ‘much broader range in the length of the stamens and staminodes’ (Hoogland, 1959); it shares its range with *D. quercifolia* and *D. schlechteri* occurs at higher altitudes. *D. insularum* is closely related to *D. nalagi* (Hoogland, 1959). *D. cyclopensis* is related to *D. papuana*, *D. quercifolia*, and *D. schlechteri* (Hoogland, 1959). *D. quercifolia* is described as having ‘non‐spreading’ petals in Hoogland (1959; p. 587). One specimen of *D. borneensis* with different stamens was believed to be a different species. Hoogland (1952) also listed 3 doubtful species, 8 excluded species and 13 invalidly published names. *D. grandifolia*, not listed here, is apetalous (Tan & Latiff, 2014).

Abbreviations: NM, not mentioned; PNG, Papua New Guinea.
